# Spatial resolution of exposure to particulate matter (PM_2.5_) and the impact on the burden of disease in Germany

**DOI:** 10.1093/eurpub/ckag048

**Published:** 2026-04-04

**Authors:** Stefan Wallek, Marcel Langner, Dietrich Plass, Sarah Kienzler, Tobias Sauter

**Affiliations:** German Environment Agency, Dessau-Roßlau, Germany; Geography Department, Faculty of Mathematics and Natural Sciences, Humboldt-Universität zu Berlin, Berlin, Germany; German Environment Agency, Dessau-Roßlau, Germany; Geography Department, Faculty of Mathematics and Natural Sciences, Humboldt-Universität zu Berlin, Berlin, Germany; German Environment Agency, Dessau-Roßlau, Germany; German Environment Agency, Dessau-Roßlau, Germany; Geography Department, Faculty of Mathematics and Natural Sciences, Humboldt-Universität zu Berlin, Berlin, Germany

## Abstract

Accurate estimation of the disease burden attributable to ambient particulate matter (PM_2.5_) requires reliable exposure data with an adequate spatial resolution. While coarse-resolution models are commonly used for national assessments, the impact of spatial resolution on environmental burden of disease (EBD) estimates remains understudied. We compared two modelling approaches for PM_2.5_ exposure in Germany: a chemical transport model (REM-CALGRID [RCG], 2 × 2 km² resolution) and a high-resolution hybrid geostatistical land-use regression model (PMR, 100 × 100 m² resolution). EBD estimates were calculated for five health outcomes using established concentration-response functions. Across all outcomes, the PMR model consistently produced slightly higher EBD estimates—approximately 2%–3% higher disability-adjusted life years (DALYs) than the coarser RCG model. The difference is due to improved detection of local pollution hotspots and more accurate exposure assignments in the high-resolution model. A larger share of the population was classified into higher exposure categories under the PMR model. Although the added value of high-resolution modelling is limited at national level, it offers clear benefits for small-area analyses and applications in environmental justice and health equity. Our findings support the integration of fine-scale exposure data into public health surveillance and burden of disease (BoD) assessments where feasible.

## Introduction

Air pollution is one of the most important global risk factors [1, 2]. For example, exposure to particulate matter (PM_2.5_) in ambient air is estimated to cause about 4.7 million deaths and contribute to 120 million disability-adjusted life years (DALYs) annually [3]. In Germany, the German Environment Agency (*Umweltbundesamt*) regularly publishes time-series analyses on the burden of disease (BoD) attributable to PM_2.5_ exposure. The current estimates present the trend of the PM_2.5_ attributable burden from 2010 to 2021. The results show a generally decreasing trend, with a reduction of about 35% from 354 748 DALYs in 2010 to 232 863 DALYs in 2021 [4].

Comparing country-specific estimates from global assessments with those derived from national level analyses reveals differences which are mainly due to different underlying assumptions [5–7]. Estimates of the BoD attributable to PM_2.5_ are the product of statistical models and rely on various input data. A key parameter is the exposure assessment, for which different methods can be used to estimate a population’s exposure towards PM_2.5_. The most common methods are chemical transport models (CTMs) considering atmospheric dispersion of pollutants and land-use regression models (LURMs) with all of them having specific advantages and limitations [8–10].

While CTMs such as REM-CALGRID (RCG) [11] are widely used in national and European air quality assessments, they typically operate on relatively coarse spatial grids. In contrast, recent advances in statistical and geospatial modelling have led to the development of high-resolution hybrid models that incorporate land use data, emission inventories, and observational measurements to improve local accuracy. However, the effect of this increased spatial granularity on environmental burden of disease (EBD) outcomes remains insufficiently explored [12–18].

This study addresses this gap by comparing EBD estimates for five health outcomes in Germany, using two different PM_2.5_ exposure models: the CTM RCG (2 × 2 km² resolution) and a hybrid land-use regression model PMR (100 × 100 m² resolution). We examine how spatial resolutions affect the distribution of exposure, the number of people classified in different concentration categories, and the resulting disease burden estimates. Particular attention is directed to the implications for small-area health analysis and environmental justice research.

## Methods

In Germany, current calculation of the EBD attributable to ambient PM_2.5_ concentration are based on outputs provided by the German Environment Agency from the RCG model [4]. RCG is a chemistry transport model designed to simulate air quality across both regional and urban scales. It integrates the regional REM3 model with the urban-scale CALGRID, operating a flexible Eulerian grid framework to capture spatial variations in pollutant concentrations.

This modelling framework supports applications across a range of spatial resolutions, typically ranging from 10 to 50 km for regional studies and 1 to 5 km for urban-scale analyses. In this study, the horizontal resolution of 2 × 2 km² was applied. The model’s nested grid capability enables high-resolution simulations within larger domains, effectively capturing pollutant transport and transformations in complex environments. RCG incorporates advanced photochemical mechanisms, as well as modules for secondary organic aerosols and sea-salt emissions. In operational use, the raw model output is corrected using interpolated concentration fields derived from approximately 250 air quality monitoring stations from the official monitoring networks of the German states and the federal government.

Meteorological inputs, including wind, temperature, and precipitation, were provided by the COSMO-DE2 model operated by the German Meteorological Service (*Deutscher Wetterdienst*), supplying high-resolution meteorological input for regional air quality simulations. RCG has been widely employed in European air quality assessments, demonstrated strong performance in capturing seasonal and spatial variability in ozone and PM_2.5_ concentrations [19–21].

In this study, an alternative dataset of PM_2.5_ concentrations was generated for 2021 using a hybrid geostatistical land-use regression model, referred to as PMR and described previously by Wallek *et al.* [22]. This model was applied to improve EBD calculations and to analyse the effects of spatial resolution on the resulting estimates.

The hybrid geostatistical approach combines Ordinary Kriging and land-use regression methods to predict PM_2.5_ concentrations across Germany at a horizontal grid resolution of 100 × 100 m. Key inputs to the model include the CORINE land cover dataset [23], Germany’s national emission inventory, a digital elevation model, and hourly PM_2.5_ measurements from 247 air quality monitoring stations operated within the official German air quality monitoring networks.

This integration of multiple data sources ensures a detailed and comprehensive representation of air quality patterns in both urban and rural settings. This model offers a high-resolution alternative to traditional CTMs such as REM-CALGRID (RCG), enabling detailed spatial analyses of air pollution exposure and its implications for health.

The analysis covers five health outcomes: chronic obstructive pulmonary disease (COPD), type 2 diabetes mellitus, ischemic heart disease, lung cancer, and stroke. For each outcome, we report the number of attributable deaths, years of life lost (YLLs), years lived with disability (YLDs), and DALYs, along with their 95% confidence intervals (lower and upper bounds, CI).

Mortality data were obtained from the official German cause-of-death statistics provided by the Federal Statistical Office of Germany (*Statistisches Bundesamt*) [24], based on ICD-10 coded death records. Morbidity data were derived from nationally representative health surveys and registry data, including the German Health Update (GEDA) studies conducted by the Robert Koch Institute [25] and cancer prevalence statistics from the German Centre for Cancer Registry Data (*Zentrum für Krebsregisterdaten*).

Population exposure was quantified using the official gridded population dataset of the Federal Statistical Office of Germany (*Statistisches Bundesamt*), based on the 2022 census and provided at a grid resolution of 1 × 1 km² [26].

## Results

**Figure 1.**
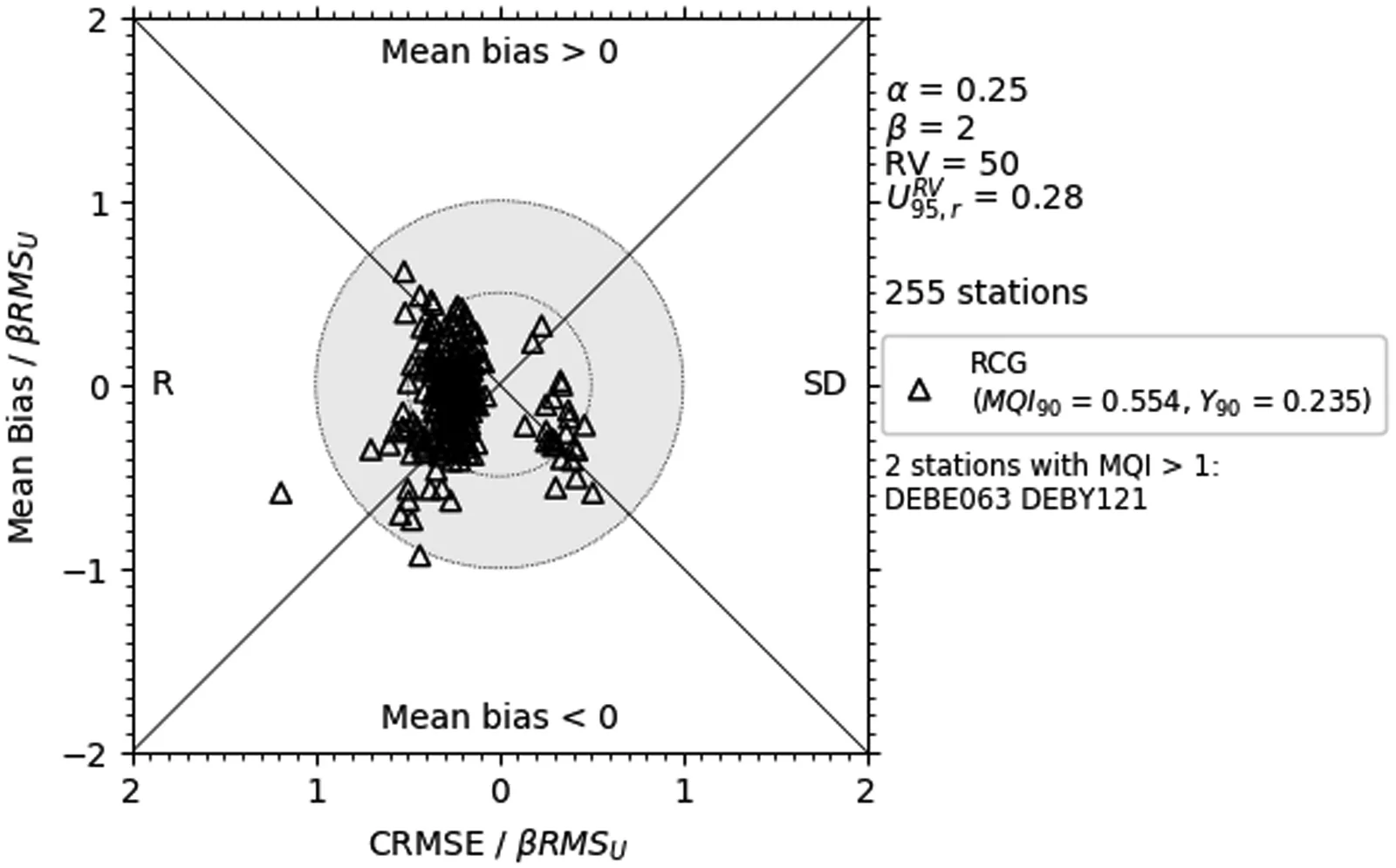
Model quality indicator (MQI) for the REM-CALGRID (RCG) model based on comparison with observed PM_2.5_ concentrations at monitoring stations across Germany. MQI values below 1 indicated by the circle depict compliance with model quality objectives according to the Delta Tool methodology.

**Figure 2.**
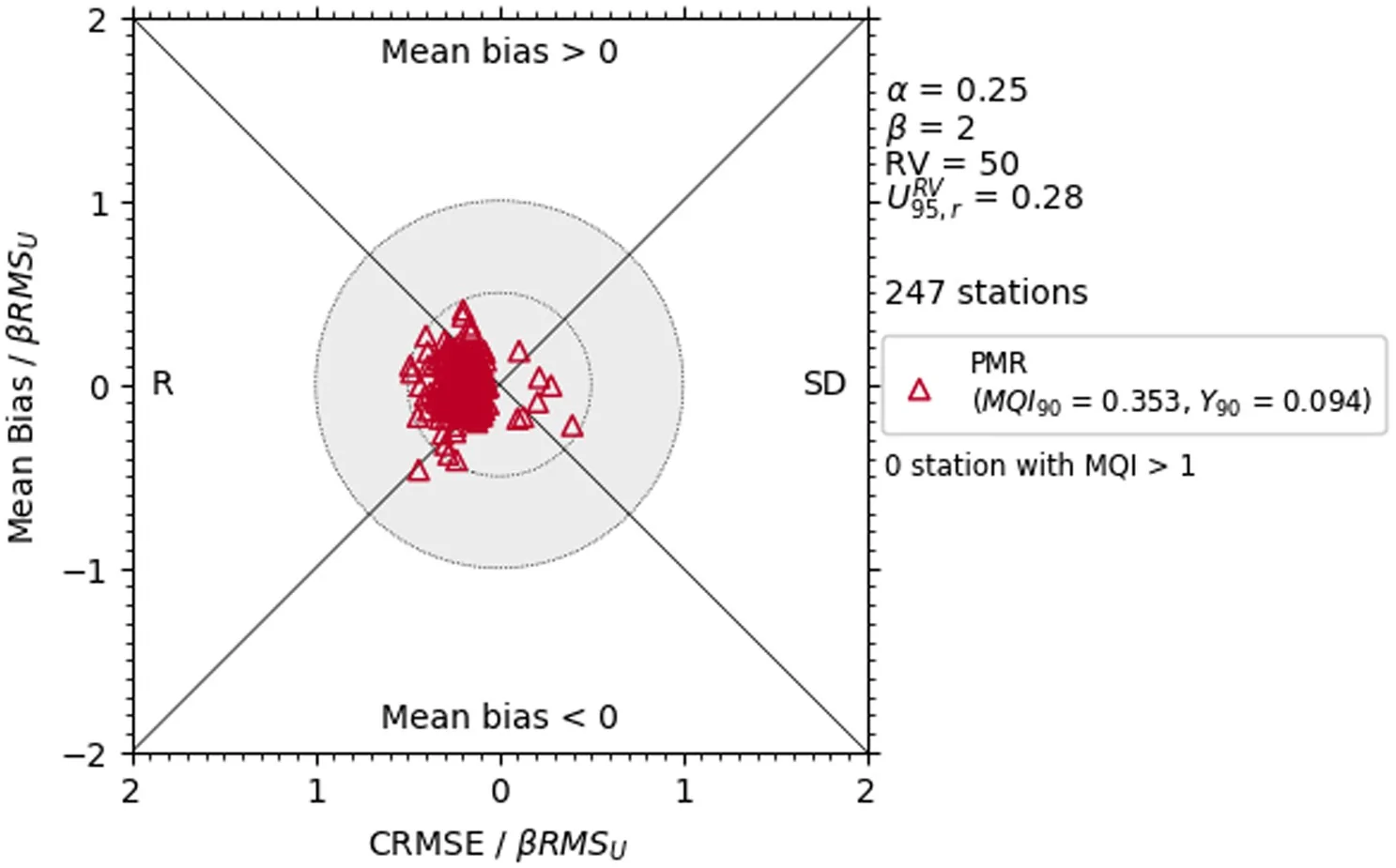
Model quality indicator (MQI) for the PMR model based on comparison with observed PM_2.5_ concentrations at monitoring stations across Germany. MQI values below 1 indicated by the circle depict compliance with model quality objectives according to the Delta Tool methodology.

Figures 1 and 2 display the model performance evaluation based on the model quality indicator (MQI), as recommended by the Delta Tool for air quality model assessment [27]. The MQI compares modelled and observed PM_2.5_ concentrations, capturing both accuracy and precision. MQI values below 1 indicate that the model meets the quality objective defined by the European Commission.

For the RCG model (Fig. 1), the majority of monitoring stations yield MQI values below 1, indicating that the model is generally in compliance with quality objectives. However, there is moderate variability across stations, and two stations exceed the MQI threshold. The model’s overall MQI is 0.554.

In contrast, the PMR model (Fig. 2) shows overall lower MQI values with a greater consistency across monitoring sites. MQI values are well below 1 at all stations, indicating a good agreement with observed concentrations and higher spatial representativeness compared to the RCG model. The model’s overall MQI is 0.353. This improved performance likely reflects the model’s integration of high-resolution land-use and emission data, which improves the model’s ability to capture local pollution dynamics.

For the direct comparison of model performance, all types of monitoring stations were included in this analysis. In operational use, however, the RCG model relies solely on background stations and therefore provides background concentrations only. In contrast, the PMR model incorporates data from all station types, including those located in industrial and traffic-related environments. When limiting the evaluation to background stations only, both models achieve MQI values below 1 at all stations. In this case, the overall MQI for the RCG model is 0.484 and 0.359 for the PMR model.

**Figure 3.**
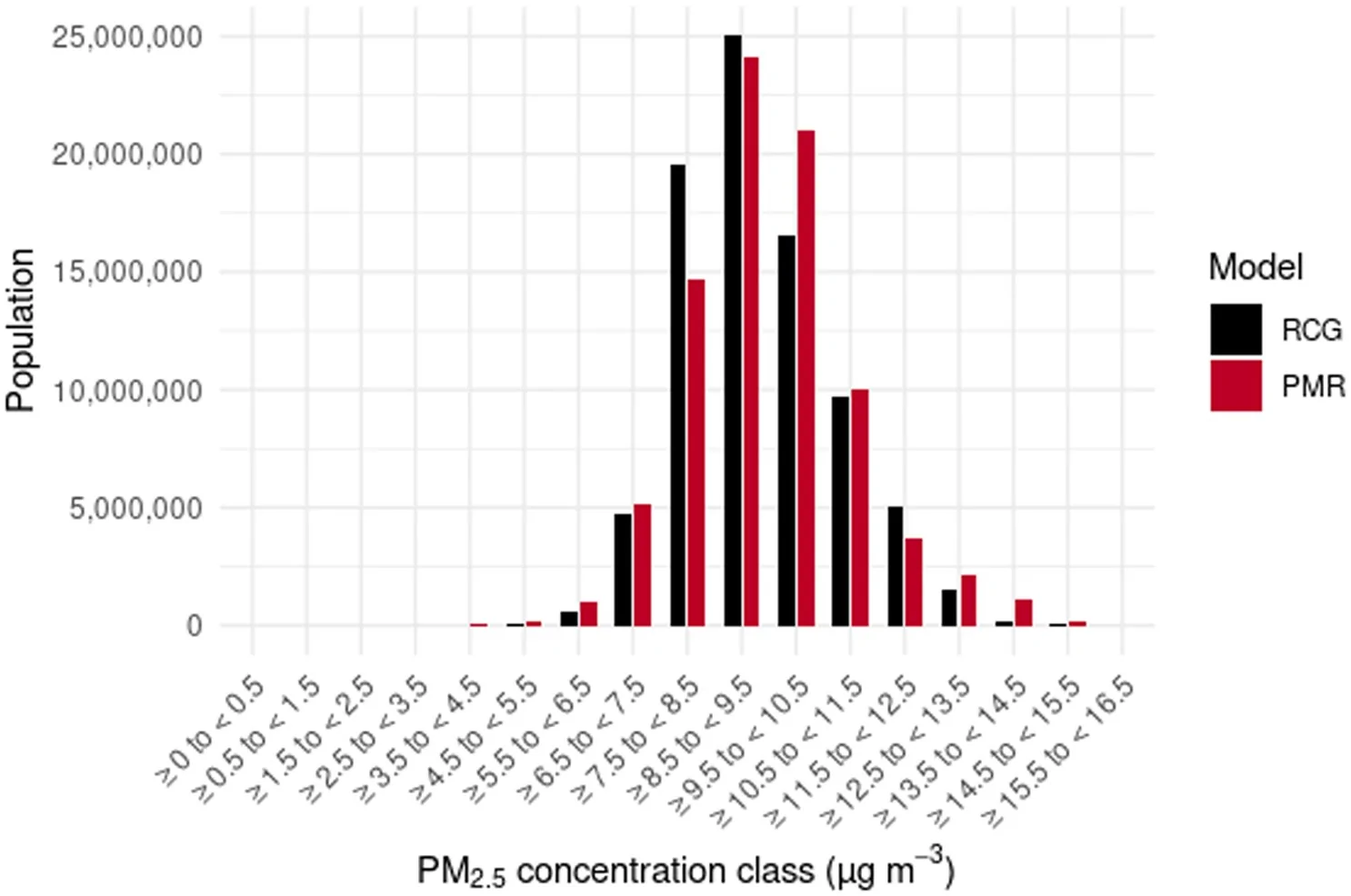
Population distribution by annual mean PM_2.5_ concentration classes based on the RCG and PMR models.

Figure 3 compares the distribution of the German population exposed to different levels of annual mean PM_2.5_ concentrations, as estimated by the RCG and PMR models. The figure highlights how population counts within specific concentration classes shift depending on the spatial resolution and methodology of the exposure model.

Overall, the PMR model classifies a larger share of the population into higher PM_2.5_ concentration classes compared to the RCG model.

Table 1 presents the EBD attributable to ambient PM_2.5_ exposure in Germany in 2021, as estimated by the exposure models.

**Table 1.** Environmental burden of disease (EBD) attributable to ambient PM_2.5_ exposure in Germany, estimated using two exposure models: the REM-CALGRID (RCG) chemical transport model and a high-resolution hybrid land-use regression model (PMR)

	RCG			PMR			RCG-PMR	
	Mean	Low	High	Mean	Low	High	Mean	%
	Chronic obstructive pulmonary disease (COPD)							
Attributable deaths	1572	1097	2064	1612	1126	2114	−40	−2.47
YLLs	19 438	13 562	25 509	19 931	13 918	26 132	−493	−2.47
YLDs	13 770	9608	18 071	14 119	9860	18 513	−349	−2.47
DALYs	33 208	23 170	43 580	34 050	23 778	44 645	−842	−2.47
	Type 2 diabetes mellitus							
Attributable deaths	1984	1287	2495	2026	1317	2541	−42	−2.06
YLLs	19 822	12 854	24 926	20 240	13 157	25 386	−418	−2.06
YLDs	34 526	22 390	43 416	35 254	22 917	44 217	−727	−2.06
DALYs	54 349	35 244	68 342	55 494	36 075	69 603	−1145	−2.06
	Ischaemic heart disease							
Attributable deaths	5354	3245	7819	5495	3336	8011	−140	−2.55
YLLs	64 352	39 059	93 309	66 131	40 229	95 706	−1779	−2.69
YLDs	5845	3550	8476	6005	3656	8693	−161	−2.68
DALYs	70 197	42 609	101 785	72 136	43 885	104 399	−1939	−2.69
	Lung cancer							
Attributable deaths	2556	1807	3347	2617	1852	3426	−61	−2.34
YLLs	40 678	28 763	53 275	41 654	29 478	54 532	−977	−2.34
YLDs	1692	1196	2215	1732	1226	2268	−41	−2.34
DALYs	42 369	29 959	55 490	43 386	30 704	56 799	−1017	−2.34
	Stroke							
Attributable deaths	1352	938	1911	1388	965	1957	−37	−2.64
YLLs	18 271	12 680	25 548	18 779	13 057	26 176	−508	−2.71
YLDs	14 469	10 054	20 138	14 865	10 348	20 625	−396	−2.66
DALYs	32 740	22 734	45 685	33 644	23 405	46 801	−904	−2.69
	Summary							
Attributable deaths	12 818	8374	17 637	13 139	8596	18 049	−320	−2.44
YLLs	162 561	106 919	222 567	166 735	109 840	227 932	−4174	−2.50
YLDs	70 302	46 798	92 317	71 976	48 007	94 315	−1673	−2.33
DALYs	192 185	124 953	261 609	197 056	128 368	267 716	−4871	−2.47

Results are shown for five health outcomes as attributable deaths, years of life lost (YLLs), years lived with disability (YLDs), and disability-adjusted life years (DALYs). Mean estimates are presented together with lower and upper uncertainty bounds (‘low’ and ‘high’). The columns RCG–PMR report absolute and relative differences between the two modelling approaches.

Across all health outcomes, the PMR-based estimates indicated consistently higher health burdens compared to those derived from the RCG model. This pattern is evident across all metrics (deaths, YLLs, YLDs, DALYs) and was most pronounced in the DALYs. For example, in the case of ischemic heart disease, the mean DALYs increased from 70 197 (95% CI: 42 609–101 785) with RCG to 72 136 (43 885–104 399) with PMR, representing an absolute difference of 1939 DALYs (+2.69%). Comparably, for stroke, the increase was 904 DALYs, also amounting to +2.69% in relative terms.

COPD showed a mean DALY increase from 33 208 (23 170–43 580) to 34 050 (23 778–44 645), and lung cancer from 42 369 (29 959–55 490) to 43 386 (30 704–56 799). Even for type 2 diabetes mellitus, the smallest relative change among all outcomes, the PMR model yielded 1145 higher DALYs than RCG (+2.06%).

The consistency of these increases across health outcomes is also evident in the summary: the total number of DALYs rose from 192 185 (124 953–261 609) in the RCG scenario to 197 056 (128 368–267 716) in the PMR scenario—an increase of 4871 DALYs or +2.47%. Notable, the upper and lower bounds of the CI also shifted slightly higher with the PMR model, though the overall width of the intervals remained relatively stable.

Taken together, these results suggest that using exposure data with finer spatial resolution leads to systematically higher estimates of the EBD, even though the relative differences remain modest (around 2%–2.7%). These differences are relevant for public health reporting and policy evaluation, as they indicate a potential underestimation of disease burden when using coarser exposure models.

## Discussion

This study assessed the impact of spatial resolution in the PM_2.5_ exposure assessment on estimates of the EBD in Germany in 2021. By comparing two modelling approaches—the chemistry transport model RCG with a resolution of 2 × 2 km² and a high-resolution hybrid land-use regression model PMR with 100 × 100 m² resolution—we found that the higher-resolution model systematically resulted in higher EBD estimates for all five considered health outcomes.

In summary, the MQI analysis confirms the higher accuracy of the PMR model in reproducing observed PM_2.5_ levels across Germany. These findings support the use of high-resolution exposure data in applications requiring precise spatial allocation of environmental health risks. It should be noted that the MQI evaluation is based on measurements from all types of air quality monitoring stations, including background, traffic, and industrial sites.

A likely explanation for this difference lies in the ability of high-resolution exposure models to better capture spatial variability, especially in densely populated urban areas with strong local emission sources [28]. The PMR model integrates detailed land use data, emission inventories, and elevation information, allowing to reflect localized pollution hotspots more accurately than coarser models [29]. Consequently, high-resolution approaches may reduce exposure misclassification—especially underestimation in highly polluted areas—which in turn leads to slightly higher estimates of disease burden.

At the population level, this implies that the high-resolution PMR approach more effectively captures small-scale pollution hotspots, particularly in urban and densely populated areas. In contrast, the RCG model tends to smooth out local variations due to its coarser 2 × 2 km² grid, leading to a greater share of the population being attributed to mid-range exposure levels.

This shift in population exposure profiles helps to explain the systematically higher burden estimates derived from the PMR model. In particular, more individuals are classified as living in environments with elevated PM_2.5_ levels, thereby increasing the population attributable fraction (PAF) and the resulting disease burden.

Although the relative differences in DALYs across health outcomes ranged between 2.06% and 2.69%, these changes are not negligible in the context of national health reporting. For instance, the overall increase of nearly 5000 DALYs due to the use of the PMR model corresponds to a substantial number of life years lost or lived with disability at population level. These differences can have implications for health policy prioritization, environmental justice considerations, and resource allocation [30–32].

However, when considering the aggregated nationwide burden estimates, the added value of higher spatial resolution is negligible. The modest overall differences suggest that for national-level assessments and trend analyses, coarser exposure models may still be sufficient. Nevertheless, the benefits of high-resolution exposure data become particularly evident in applications that rely on small-area analyses [33]. For example, these include studies on environmental justice, health disparities, or local public health interventions. At such scales, detailed exposure data can be meaningfully combined with socio-demographic or contextual information—such as indicators of socioeconomic status, education, income, or migration background—often available at neighbourhood or census tract level. This enables targeted analyses of vulnerable population groups and supports the design of tailored, equity-oriented health policies and communication strategies.

Beyond the specific national case study presented here, the results also highlight the potential relevance of high-resolution exposure modelling for regions with limited data availability. In settings where monitoring networks are sparse or unevenly distributed, fine-scale exposure models that integrate auxiliary data sources—such as land use, emissions, and topography—may help to reduce spatial data gaps and improve population exposure estimates. In such contexts, high-resolution approaches can complement coarser models by providing a more differentiated representation of exposure patterns, even when dense measurement data are not available. This may enhance the applicability of BoD assessments in regions facing substantial spatial heterogeneity and data limitations.

Our findings are consistent with previous studies emphasizing the importance of spatial resolution in environmental epidemiology and BoD modelling. Studies employing finer-scale exposure assessments often report higher risk estimates, particularly in urban contexts where average values from coarse models may mask intra-urban heterogeneity [7, 33–35]. These results reinforce the view that improving the spatial resolution of exposure estimates can enhance the accuracy and granularity of EBD assessments, especially in equity-sensitive contexts.

Taken together, these results suggest that using exposure data with finer spatial resolution leads to systematically higher estimates of the EBD, even though the relative differences remain modest (around 2%–2.7%). These differences are relevant for public health reporting and policy evaluation, as they indicate a potential underestimation of disease burden when using coarser exposure models.

For potential future applications, the PMR model was further evaluated based on daily mean concentrations using leave-one-out cross-validation (*n* = 93 884). This additional evaluation was conducted to assess the robustness of the exposure estimates for subsequent use beyond the present analysis. Although an evaluation based on hourly means would offer finer temporal resolution, it is considerably more resource-intensive and is unlikely to yield substantially improved results, given the high autocorrelation of hourly PM_2.5_ measurements (r = 0.66 at lag 24).

The cross-validation resulted in the following performance metrics: correlation = 0.93, mean absolute error (MAE) = 1.61 µg m^−^³, root mean square error (RMSE) = 2.31 µg m^−^³, bias (observed–predicted) = 0.05 µg m^−^³, relative bias = −0.05, and relative absolute bias = 0.23.

Nonetheless, several limitations must be acknowledged. First, the underlying exposure models do not only differ in spatial resolution but also in methodology, which may account for the differences in health estimates [36]. While PMR incorporates empirical ground-based measurements and geostatistical methods, RCG relies on simulated atmospheric processes, including long-range pollutant transport. Thus, part of the observed differences may reflect model structure rather than resolution alone. However, it is noteworthy that the relative differences between models remained consistent across all health outcomes [37].

Future research should explore hybrid approaches that combine the strengths of both model types and systematically quantify the effects of spatial resolution across other pollutants and health outcomes. In particular, integrating exposure models with high spatial resolution into local or urban health surveillance systems could improve the identification of pollution-related health inequalities and help raise awareness for environmental justice at the community level. Higher spatial resolution is also beneficial for other air pollutants, particularly those more strongly influenced by highly localized conditions—such as nitrogen dioxide (NO_2_) emissions from traffic—than PM_2.5_ [34, 38, 39].

In summary, our findings suggest that while high-resolution exposure models yield slightly higher estimates of PM_2.5_-attributable disease burden, the added value at national level remains modest. However, for small-area analyses and applications in environmental justice or health equity, the use of detailed exposure data offers significant benefits. To support more public health decision-making and address spatial health inequalities, future BoD assessments should consider integrating high-resolution exposure modelling where feasible and contextually relevant.

## Data Availability

The data presented in this study are available upon request from the corresponding author. The data are not publicly available due to their ownership to the states and the federal government. Access to these datasets necessitates a formal request. Key Points Higher spatial resolution of PM_2.5_ exposure leads to higher estimates of disease burden. Differences between fine- and low-resolution exposure models are modest at national level but consistent across health outcomes. Fine-scale exposure models better capture population exposure in densely populated areas. Model structure, in addition to spatial resolution, contributes to differences in burden estimates.
