# Correction to: Spatial resolution of exposure to particulate matter (PM_2.5_) and the impact on the burden of disease in Germany

**DOI:** 10.1093/eurpub/ckag170

**Published:** 2026-09-24

This is a correction to: Stefan Wallek, Marcel Langner, Dietrich Plass, Sarah Kienzler, Tobias Sauter, Spatial resolution of exposure to particulate matter (PM_2.5_) and the impact on the burden of disease in Germany, *European Journal of Public Health*, Volume 36, Issue 2, April 2026, ckag048, https://doi.org/10.1093/eurpub/ckag048

In the originally published version of this manuscript, the graphs of Figures 2 and 3 were erroneously switched.

This error has been corrected.

